# Lanthanum-Doped Co_3_O_4_ Nanocubes Synthesized via Hydrothermal Method for High-Performance Supercapacitors

**DOI:** 10.3390/nano15191515

**Published:** 2025-10-03

**Authors:** Boddu Haritha, Mudda Deepak, Merum Dhananjaya, Obili M. Hussain, Christian M. Julien

**Affiliations:** 1 Thin Films Laboratory, Physics Department, Sri Venkateswara University, Tirupati 517502, India; harithathinfilms@gmail.com (B.H.); deepakmudda7@gmail.com (M.D.); msdhananjaya51@gmail.com (M.D.); 2 Institut de Minéralogie, de Physique des Matériaux et Cosmologie (IMPMC), Sorbonne Université, UMR-CNRS 7590, 4 Place Jussieu, 75252 Paris, France

**Keywords:** lanthanum doping, spinel oxide, cobalt oxide nanocubes, hydrothermal synthesis, supercapacitors

## Abstract

The development of high-performance supercapacitor electrodes is crucial to meet the increasing demand for efficient and sustainable energy storage systems. Cobalt oxide (Co_3_O_4_), with its high theoretical capacitance, is a promising electrode material, but its practical application is hindered by poor conductivity limitations and structural instability during cycling. In this work, lanthanum La^3+^-doped Co_3_O_4_ nanocubes were synthesized via a hydrothermal approach to tailor their structural and electrochemical properties. Different doping concentrations (1, 3, and 5%) were introduced to investigate their influence systematically. X-ray diffraction confirmed the retention of the spinel phase with clear evidence of La^3+^ incorporation into the Co_3_O_4_ lattice. Also, Raman spectroscopy validated the structural integrity through characteristic Co-O vibrational modes. Scanning electron microscopy analysis revealed uniform cubic morphologies across all samples. The formation of the cubic spinel structure of 1% La^3+^-doped Co_3_O_4_ are confirmed from XPS and TEM studies. Electrochemical evaluation in a 3 M KOH electrolyte demonstrated that 1% La^3+^-doped Co_3_O_4_ nanocubes delivered the highest performance, achieving a specific capacitance of 1312 F g^−1^ at 1 A g^−1^ and maintaining a 79.8% capacitance retention and a 97.12% Coulombic efficiency over 10,000 cycles at 5 Ag^−1^. It can be demonstrated that La^3+^ doping is an effective strategy to enhance the charge storage capability and cycling stability of Co_3_O_4_, offering valuable insights for the rational design of next-generation supercapacitor electrodes.

## 1. Introduction

The rising global demand for energy, coupled with growing environmental concerns, has placed immense pressure on the search for efficient, sustainable, and environmentally benign energy storage technologies. The depletion of fossil fuel reserves and the release of greenhouse gases are exacerbating these challenges, thereby intensifying research into alternative electrochemical systems for energy storage and conversion [1,2,3]. Among the wide range of candidates, including capacitors, batteries, and fuel cells, supercapacitors have emerged as a promising class of devices. They bridge the performance gap between traditional capacitors and batteries by offering both high power density and relatively high energy density. The performance of supercapacitors strongly depends on both material and operational factors, including the electrode–electrolyte interface, electrical contact with the current collector, operating temperature, and the applied current [4,5]. In recent decades, they have attracted considerable attention due to their advantageous features, including fast charge–discharge kinetics, high Coulombic efficiency, long cycle life, and cost-effective fabrication. Based on their charge storage mechanisms, supercapacitors are broadly categorized as: (i) electric double-layer capacitors (EDLCs), which rely on carbon-based nanostructures such as activated carbon, graphene, and carbon nanotubes, and (ii) pseudocapacitors, which utilize transition-metal oxides (TMOs) such as RuO_2_, NiO, Fe_3_O_4_, Mn_3_O_4_, V_2_O_5_, and Co_3_O_4_ materials to store charge through rapid and reversible Faradaic reactions [6,7,8,9,10,11,12].

Co_3_O_4_ nanoparticles have garnered considerable research interest among metal oxide nanoparticles due to their affordability and superior electroactivity. Co_3_O_4_ being a transition metal oxide, can display various oxidation states (Co^2+^, Co^3+^, and Co^4+^), enhancing its complex redox chemistry. Co_3_O_4_ is a multifunctional, antiferromagnitic p-type semiconductor characterized by a spinel crystal structure. The direct optical band gaps of Co_3_O_4_ nanoparticles are approximately 1.48 and 2.19 eV, facilitating their applications in several domains including catalysis [13], gas detection [14], water splitting [15], and biological [16] uses. Furthermore, Co_3_O_4_ has arisen as a viable electrode material for electrochemical energy storage systems, especially supercapacitors, due to its significant theoretical specific capacity of approximately 3560 F g^−1^ and the availability of numerous redox active sites that promote rapid and reversible faradaic reaction. However, its practical application is hindered by poor intrinsic conductivity, high internal resistance, and a relatively small electroactive surface area, resulting in capacitances that are far below the theoretical value [12,17,18]. To overcome these limitations, two primary strategies have been widely explored: (1) morphology engineering to expose more active sites, and (2) cation doping to improve conductivity and electrochemical activity [19,20]. An assortment of dopants such as Ni, Mn, Fe, Cu, Zn, and Cr has been integrated into the Co_3_O_4_ lattice to lower resistance and create more redox-active sites [21,22,23,24,25,26,27]. Additionally, Co_3_O_4_-based composites combined with carbon materials, conducting polymers and other metal compounds significantly improve its electrochemical performance for energy storage applications. For example, Annu et al. summarized that cobalt-based nanocomposite combined with conducting polymers such as polypyrrole (PPy) and polyaniline (PANI) exhibits superior electrochemical performance owing to the synergistic effect of cobalt oxide’s high capacitance and the polymers conductivity and pseudocapacitive behavior, making them promising for use in supercapacitors, batteries, and hybrid devices [28]. Deng et al. incorporated cadmium into porous Co_3_O_4_ nanosheets through a co-precipitation approach, and the optimized composition with 5% Cd delivered a specific capacitance of 737 F g^−1^at 1 A g^−1^, retaining 96% of its capacity after 1000 cycles [29]. Similarly, chromium-doped Co_3_O_4_ nanoflowers synthesized by Faisal et al. via a hydrothermal method exhibited a maximum capacitance of 1283 F g^−1^ at 6 at.% Cr and maintained 72.86% of their initial capacitance after 1000 charge–discharge cycles [2]. Aadil et al. reported the growth of mesoporous Ag-doped Co_3_O_4_ nanosheets on nickel foam, which offered a large surface area (176 m^2^ g^−1^) and excellent electrochemical properties, including 1425 F g^−1^at 1 A g^−1^, 92.84% retention at 10 A g^−1^, and stable cycling with 96.4% retention over 5000 cycles [30]. Additionally, Ce-doped Co_3_O_4_ nanoflakes were produced by Faisal et al. via the hydrothermal method and demonstrated an optimal performance at 5.0 at.% Ce, yielding 1309.6 F g^−1^ at 5 mV s^−1^, with 90.86% capacitance retention after 2000 cyclic voltammograms, and a rate capability of 82.87% at 10 A g^−1^ [31]. Furthermore, Aslam et al. incorporated Ni, Mn, and Zn dopants into Co_3_O_4_ via a co-precipitation process, where Mn_0.05_Co_2.95_O_4_ exhibited the most promising pseudocapacitive behavior with 80.8 F g^−1^ at 1 A g^−1^ along with excellent cycling stability [32].

More recently, rare earth metal doping (La, Nd, Gd, and Sm) has been investigated as a promising approach to further enhance the electrical conductivity and supercapacitor performance of Co_3_O_4_ [33]. In particular, Sm-doped Co_3_O_4_ electrodes exhibited excellent performance with a capacitance retention of 93.18%. The high ionic radii and strong complexation ability of rare earth ions can induce lattice distortion, resulting in smaller crystallite sizes, enhanced electrical conductivity, and reduced charge transfer resistance. Specifically, La^3+^ ions (with an ionic radius of 1.16 Å) exhibit a strong affinity for oxygen-containing functional groups, which stabilizes the surface structure and enhances electrical conductivity when incorporated into the Co_3_O_4_ [34]. In addition, various synthesis strategies have been explored for the fabrication of nanostructured Co_3_O_4_, including sol–gel, spray pyrolysis, chemical deposition, solvothermal, co-precipitation, and hydrothermal processes. Among these, the hydrothermal method stands out due to its simplicity, scalability, and precise control over morphology through reaction parameters (temperature, duration, precursor concentration) [34,35,36,37,38,39,40,41].

Therefore, in this study, La-doped Co_3_O_4_ nanocubes were synthesized via a facile hydrothermal method with varying La concentrations (1–5 mol%). The impact of La doping on the structural, morphological, and electrochemical properties was systematically evaluated. The results demonstrate that incorporating 1 mol% La significantly enhances the electrochemical performance, establishing La-doped Co_3_O_4_ as a highly promising electrode material for next-generation supercapacitor applications.

## 2. Materials and Methods

### 2.1. Chemical Materials

All chemicals and solvents used in this work were of reagent grade and employed without further purification. Cobalt (II) nitrate hexahydrate (Co(NO_3_)_2_·6H_2_O), lanthanum (III) nitrate hexahydrate (La(NO_3_)_3_·6H_2_O, 99.99%), and polyvinylidene fluoride (PVDF) were purchased from Sigma-Aldrich (Saint-Louis, MS, USA). Potassium hydroxide (KOH) pellets were obtained from Molychem. Additional materials included deionized (DI) water, ethanol (99.5%), activated carbon, and N-Methyl-2-pyrrolidone (NMP).

### 2.2. Preparation of Lanthanum-Doped Co_3_O_4_ Nanoparticles

**Figure 1:**
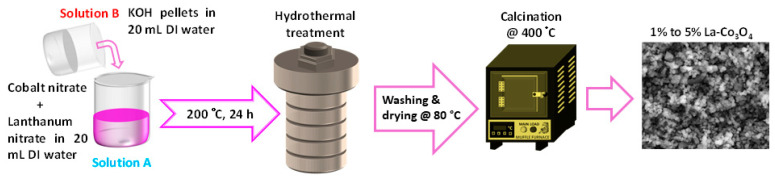
Schematic representation of the synthesis procedure for the preparation of La (1–5%) doped cobalt oxide nanocubes.

Lanthanum-doped Co_3_O_4_ nanoparticles were synthesized via a hydrothermal method, as illustrated in Figure 1. Solution A was prepared by dissolving 1 mol L^−1^ Co(NO_3_)_2_·6H_2_O in 20 mL of DI water under constant stirring. Lanthanum nitrate was added in molar ratios of 1%, 3%, and 5% with respect to Co^2+^ ions, and the solution was until complete dissolution. Separately, solution B was prepared by dissolving 1 mol L^−1^ KOH in 20 mL of DI water. The solution was subsequently added drop wise to solution A while continuously stirring to achieve a homogenous mixture, with the pH of the combined solution maintained at 9 to ensure regulated nucleation and development of the nanoparticles. The resulting solution was transferred into a 100 mL Teflon-lined stainless-steel autoclave and heated at 200 °C for 24 h. After cooling to room temperature. The precipitated was collected, washed four times with ethanol and deionized water in order to remove impurities, and afterwards dried at 80 °C for 12 h. The dried powder was then calcinated at 400 °C for 4 h, utilizing a controlled heating rate of 3 °C min^−1^. The final products were labeled as 1%, 3%, and 5% La-Co_3_O_4_, reflecting the different lanthanum contents.

### 2.3. Methods

The microstructural properties of the Co_3_O_4_ nanocubes were characterized by X-ray diffraction (XRD, Miniflex600, Rigaku, Tokyo, Japan) using CuK_α_ radiation (λ = 1.5406 Å) as X-ray source. Surface morphology was examined using a field emission scanning electron microscope (FESEM) (Carl Ziess, Jena, Germany) and a high-resolution transmission electron microscope (HR-TEM JEM-2100, JEOL, Tokyo, Japan). Raman spectroscopy (Renishaw, Wottenunder-Edge, UK) equipped with a 532 nm excitation laser (power of 50 mW) was employed to analyze the vibrational modes and to confirm the phase purity of the samples. The valance state and the elemental composition were analyzed with X-ray photoelectron spectroscopy (XPS, Kratos AXIS-Nova, Shimadzu, Tokyo, Japan).

The electrochemical performance of the prepared cobalt oxide electrodes was evaluated in a 3 mol L^−1^ KOH aqueous electrolyte within a voltage range of 0.0 to 0.5 V at room temperature, using a CHI 608C electrochemical workstation (CH Instruments Inc., Austin, TX, USA) in a three-electrode glass cell. The working electrode was prepared by combining 80 wt.% active material, 10 wt.% PVDF, and 10 wt.% carbon black. After grinding these components for 3 h, a small amount of NMP solution was added to form a smooth slurry. Cleaned nickel foam pieces (1.5 cm^2^) were coated with the slurry through a drop-casting technique and dried overnight at 100 °C. For electrode preparation, commercial nickel foam sheets were cut into 1.5 cm × 1 cm pieces. Some foam pieces were immersed in 3 mol L^−1^ hydrochloric acid and then sonicated for 15 min to remove any residual hydrocarbons on the surface. The cleaned foams were then rinsed with deionized water and ethanol in an ultrasonic bath and dried in a vacuum oven for later use. The electrode mass loading was ~2 mg cm^−2^. The Ag/AgCl and a platinum strip were used as reference and counter electrodes, respectively.

## 3. Results and Discussion

### 3.1. Structure

**Figure 2:**
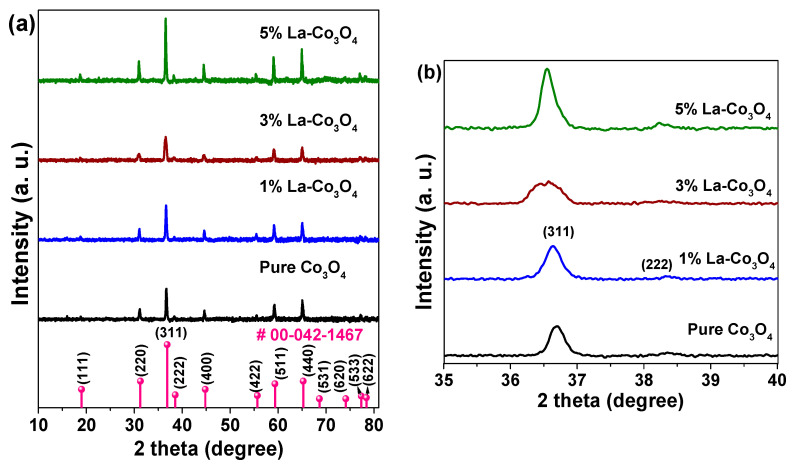
(**a**) XRD spectra of pure and La (1–5%) doped Co_3_O_4_ nanocubes recorded using a CuK*_α_* X-ray source. (**b**) Expanded view of (311) and (222) diffraction planes.

Figure 2a presents the XRD patterns of pure and La-doped Co_3_O_4_ nanocubes with varying La contents (1, 3, and 5 mol%). The diffraction peaks of pristine Co_3_O_4_ sample are well indexed to the cubic spinel phase (JCPDS No. 00-042-1467), corresponding to the (111), (220), (311), (222), (400), (422), (511), (440), (531), (620), (533), and (622) planes. This confirms the formation of a single-phase spinel structure with a space group of *Fd*3*m* [42]. The absence of additional peaks further indicates the high phase purity of the samples. For the La-doped samples, no reflections corresponding to La-based secondary phases (e.g., La_2_O_3_ or LaCoO_3_) were observed, suggesting successful substitution of La^3+^ into the Co_3_O_4_ lattice or the presence of amorphous La species below the detection limit of XRD. However, slight peak shifts, variations in relative intensities, and changes in peak broadness reveal structural modifications induced by lanthanum incorporation (Figure 2b).

At the 1 mol% La doping level, the (311) reflection exhibits slight broadening and enhanced intensity compared to the undoped sample. This is attributed to the lattice distortion and microstrain resulting from the substitution of Co^3+^ (ionic radius ~0.545 Å) with larger La^3+^ ions (ionic radius ~1.16 Å). The size mismatch introduces internal strain and crystallographic defects such as dislocations and vacancies. Increasing the La concentration to 3 mol% further broadens the peaks and decreases their intensity, implying a higher density of defects and microstrain, possibly due to excessive La incorporation that disrupts the spinel structure and limits crystallite growth. Interestingly, at 5 mol% La doping, the (311) peak becomes sharper and more intense than those of the 1% and 3% doped samples. This suggests partial recovery of crystallinity, likely due to saturation of La^3+^ solubility in the spinel lattice. At this level, La^3+^ ions may segregate toward grain boundaries or form amorphous La-rich domains undetectable by XRD, thereby relieving lattice strain and allowing coherent crystal growth, particularly along the (311) orientation.

The crystallite size (*D*) was estimated using Debye–Scherrer′s equation [43]

(1) $$D=\frac{0.94\lambda }{\beta \;cos\theta }$$

where *λ* is the X-ray wavelength (1.5406 Å for Cu K_α_), *β* is the full width at half maximum (FWHM) in radians, and *θ* is the Bragg angle of the (311) planes. The dislocation density (*δ*) and microstrain (*ε*) were calculated using the relations [44,45]

(2a) $$\delta =\frac{1}{D^{2}},$$

(2b) $$\varepsilon =\frac{\beta }{4\tan\theta }.$$

The calculated values are summarized in Table 1. Pristine Co_3_O_4_exhibits a crystallite size of 30.6 nm. At the 1% La-doping content, the size slightly increases to 32.4 nm, which may result from slight lattice expansion due to the limited La^3+^ substitution into the Co_3_O_4_ lattice. At the 3% La-doping level, the crystallite size decreases sharply to 17.5 nm, accompanied by a notable increase in dislocation density and microstrain, which confirms a greater degree of structural disorder. In contrast, at the 5% La-doping content, the crystallite size increases to 38.0 nm with a decrease in microstrain, consistent with a La segregation and a partial strain relaxation at high doping levels.

**Figure 3:**
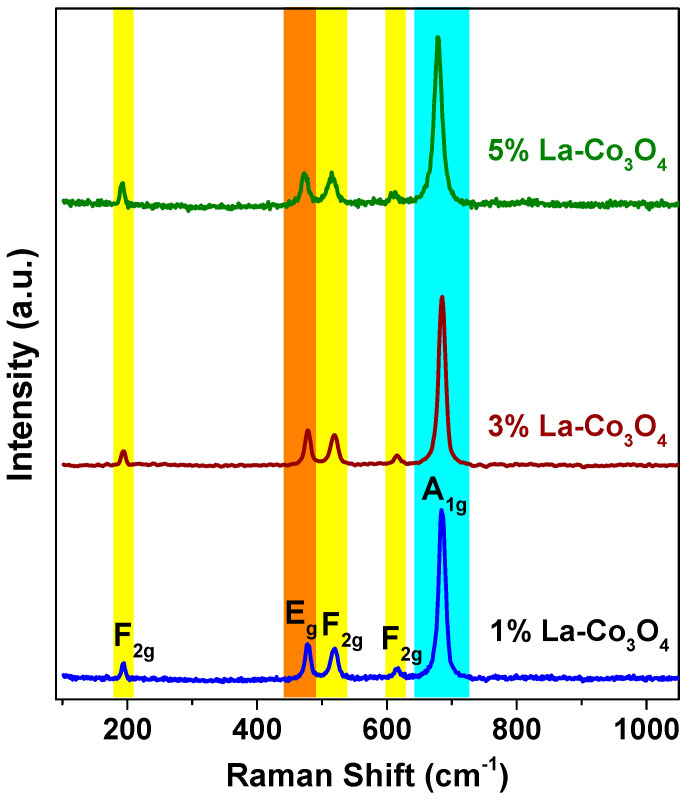
Raman spectra of La-doped (1–5%) Co_3_O_4_ nanocubes recorded with a 532 nm excitation laser (power of 50 mW).

The Raman spectra (Figure 3) of pristine and La-doped Co_3_O_4_ nanocubes exhibit five Raman-active modes at 194, 478.5, 520.1, 616.5, and 684.6 cm^−1^, corresponding to the *F*_2g_, *E*_g_, *F*_2g_, *F*_2g_, and *A*_1g_ phonon modes, respectively. These are in good agreement with the reported Raman modes of Co_3_O_4_. The band at 194 cm^−1^ arises from the translational vibration of Co^2+^–O^2−^ at tetrahedral sites, while the 684.6 cm^−1^ band corresponds to the symmetric stretching vibration of Co^3+^–O^2−^ in CoO_6_octahedra. The bands at 478.5, 520.1, and 616.5 cm^−1^ are associated with bending and stretching modes of Co–O bonds in octahedral sites. Slight shifts in peak positions with increasing La doping suggest lattice distortion and particle size effects. Notably, no Raman peaks attributed to La-based phases were detected, supporting the XRD results [46,47,48].

### 3.2. XPS Analysis

**Figure 4:**
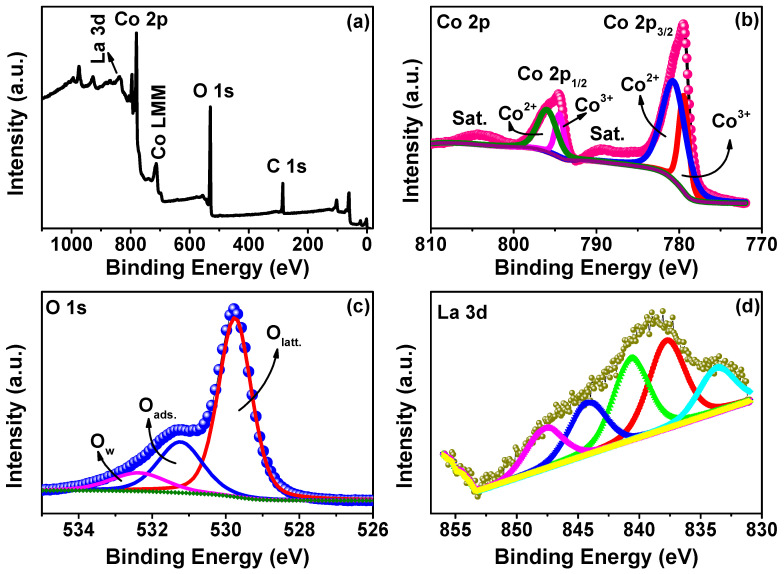
(**a**) XPS survey spectrum and high-resolution (**b**) Co 2p XPS spectrum, (**c**) O 1s XPS spectrum, and (**d**) La 3d XPS spectrum of the 1 mol% La-doped Co_3_O_4_ sample.

The surface chemical states of the 1 mol% La-doped Co_3_O_4_nanocubes were investigated using XPS experiments (Figure 4). The survey spectrum (Figure 4a) confirms the presence of Co 2p, O 1s, La 3d, and C 1s peaks, as well as minor Auger features, validating the successful incorporation of La into the cobalt oxide lattice. The high-resolution Co 2p spectrum (Figure 4b) displays two prominent peaks at 779.5 eV (Co 2p_3/2_) and 794.6 eV (Co 2p_1/2_), along with corresponding shake-up satellites. Deconvolution reveals contributions from both Co^3+^ (779.4 eV and 794.5 eV) and Co^2+^ (780.7 eV and 796.0 eV), confirming the mixed-valence state characteristic of the spinel Co_3_O_4_ phase. The presence of shake-up satellites further supports this coexistence [46,49]. The O 1s spectrum (Figure 4c) consists of three distinct components at 529.7 eV (lattice oxygen, O_latt_), 531.2 eV (surface-adsorbed oxygen species, O_ads_), and 532.4 eV (hydroxyl groups or adsorbed water molecules, O_w_) [50,51]. The dominance of the O_latt_ contribution indicates a well-ordered crystalline structure and effective oxygen incorporation within the spinel structure. The high-resolution La 3d spectrum (Figure 4d) reveals a broad spectral profile, deconvoluted into multiple peaks centered at 833.7, 837.7, 840.6, 844.2, and 847.7 eV. These values exhibit slight deviations from the standard binding energies typically associated with La^3+^ (La 3d_5/2_ at ~834 eV and La 3d_3/2_ at ~851 eV) [52]. The observed shifts and broadening may result from lattice distortions due to the low La doping level, leading to modified local coordination environments around the La ions. Quantitative XPS analysis reveals a surface La atomic percentage of 0.69%, which aligns reasonably well with the intended 1 mol% doping level, considering the surface-sensitive characteristics of XPS. The calculated La/(La + Co) atomic ratio is around 2.85, indicating a slight surface enrichment, a phenomenon commonly observed in metal oxide systems upon doping [53,54]. These results collectively confirm the successful substitution of La^3+^ into the Co_3_O_4_ lattice, likely occupying Co^3+^ sites and preserving the spinel structure.

### 3.3. Morphology

**Figure 5:**
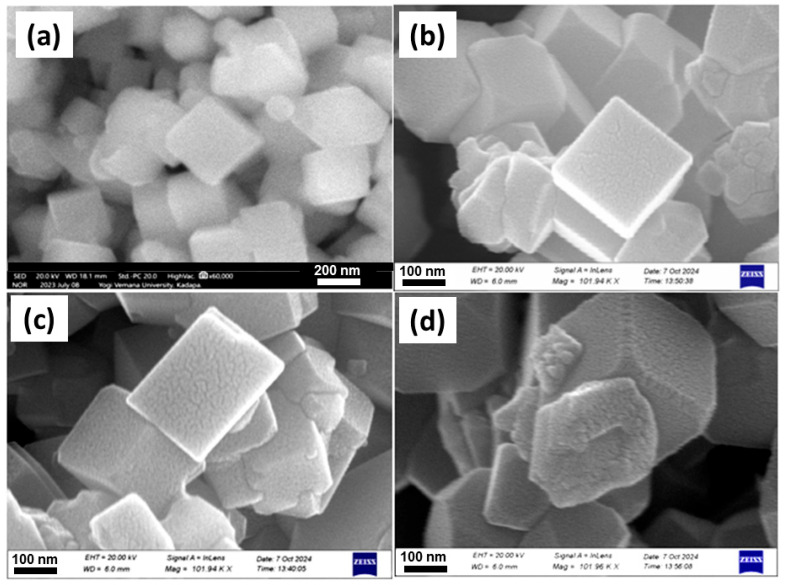
FE-SEM images of La-doped Co_3_O_4_ nanocubes synthesized via hydrothermal method: (**a**) pristine, (**b**) 1 mol%, (**c**) 3 mol%, and (**d**) 5 mol% La-doped sample.

The surface morphology of La-doped Co_3_O_4_ samples was analyzed via field-emission scanning electron microscopy, as shown in Figure 5. All samples exhibit a well-defined cubic-like morphology, typical of spinel Co_3_O_4_ nanostructures. However, the La doping concentration significantly influences the uniformity and surface texture of the resulting particles. At a low doping level of 1 mol% La (Figure 5a), the particles appear as highly uniform nanocubes with smooth surfaces and sharply defined edges. This morphology suggests a high degree of crystallinity and minimal structural distortion, both of which are beneficial for enhanced electrochemical performance due to increased surface area and improved charge transport pathways. Increasing the La content to 3 mol% (Figure 5b) largely retains the cubic morphology, but noticeable surface roughness and particle aggregation begin to emerge.

This result suggests a partial distortion of the crystal growth process, probably resulting from an excessive La incorporation, which may cause lattice strain or substitutional defects. The morphology of the 5 mol% La-Co_3_O_4_ sample (Figure 5c) exhibits a trend towards greater irregularity, characterized by less-defined cube structures and rough surfaces. The agglomeration of particles is more pronounced, and the uniformity of the nanostructures is significantly reduced. Such morphological deterioration may hinder electrochemical performance due to reduced active surface area and impaired ion/electron diffusion. These observations suggest that a low level of La doping (1 mol%) is optimal for maintaining a desirable nanocubic morphology and maximizing electrochemical performance. In contrast, higher La concentrations negatively affect particle uniformity and crystallinity, likely due to disruption of the spinel lattice during growth.

The elemental composition of the synthesized 1 mol% La-doped Co_3_O_4_ sample was analyzed with energy dispersive X-ray spectroscopy (EDS). The EDS spectrum is given as supplementary data in Figure S1 (Supplementary Materials). The presence of cobalt, oxygen, and lanthanum at their respective binding energies confirm a stoichiometric ratio consistent with the spinel Co_3_O_4_ phase with cobalt (26.59 at.%), oxygen (71.45 at.%) and lanthanum (1.96 at%) present in a stoichiometric ratio consistent with the spinel cobalt oxide (Co_3_O_4_) phase. The elemental mapping also confirms the uniform distribution of cobalt and oxygen throughout the scanning area of the nanocubes.

**Figure 6:**
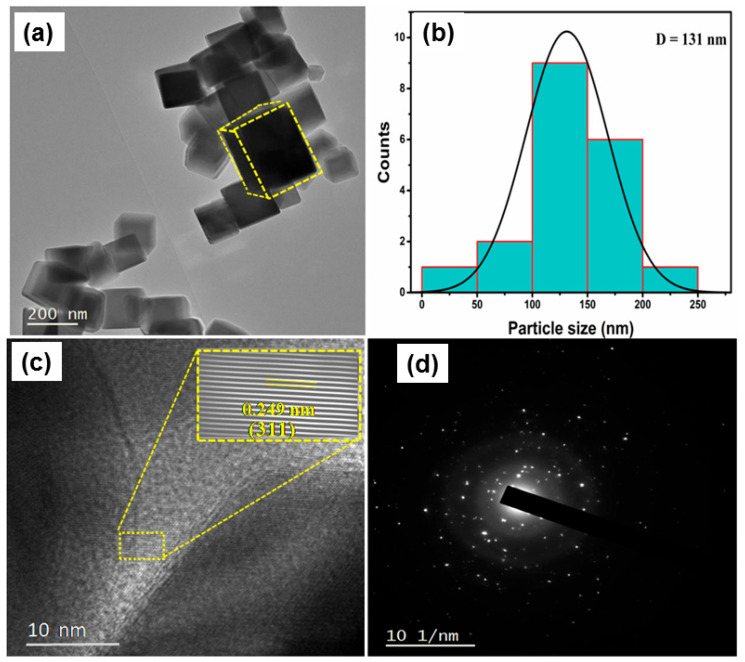
HR-TEM analysis of 1 mol% La-doped Co_3_O_4_ nanocubes: (**a**) TEM image, (**b**) histogram of particle size distribution, (**c**) HR-TEM lattice fringe image, and (**d**) SAED pattern.

High-resolution transmission electron microscopy (HR-TEM) was utilized to examine the nanostructural properties of 1% La-doped Co_3_O_4_ nanocubes. Figure 6a illustrates a TEM picture that distinctly reveals a well-defined cubic morphology with sharp edges, thereby verifying the successful synthesis of homogeneous nanocubes. The particle size distribution obtained from many TEM images (Figure 6b) reveals an average particle size of approximately 131 nm. The lattice fringes in the HR-TEM image (Figure 6c) display an interplanar spacing of 0.249 nm, corresponding to the (311) plane of 1% La-Co_3_O_4_. The FFT pattern depicted in Figure 6c further substantiates the crystallinity of the nanocubes and the existence of distinctly resolved lattice planes. The minor increase in interplanar spacing relative to pure Co_3_O_4_ is due to the integration of bigger La^3+^ ions into the spinel lattice, resulting in localized lattice strain and minor distortions while preserving the overall cubic symmetry. The selected area electron diffraction (SAED) pattern (Figure 6d) exhibits bright spots accompanied by somewhat diffused rings, signifying the well-formed crystalline character of the nanocubes with a preferential orientation along the specific crystallographic axes. The integrated HR-TEM and SAED findings align remarkably with XRD investigations, validating that La doping marginally enlarges the lattice, maintains crystallinity, and does not alter the distinctive cubic shape of Co_3_O_4_. These structural alterations are anticipated to improve electrochemical characteristics by increasing active sites and enhancing ion transport channels within the nanocubes.

### 3.4. Electrochemical Properties

The electrochemical properties of La-doped Co_3_O_4_ electrodes were systematically studied using electrochemical impedance spectroscopy (EIS), cyclic voltammetry (CV), and galvanostatic charge–discharge (GCD) in a three-electrode configuration. The working electrodes consisted of La-doped Co_3_O_4_ composites with 1%, 3%, and 5% La content, while Ag/AgCl and Pt foil were used as the reference and counter electrodes, respectively. All measurements were carried out in a 3 mol L^−1^ KOH aqueous electrolyte.

**Figure 7:**
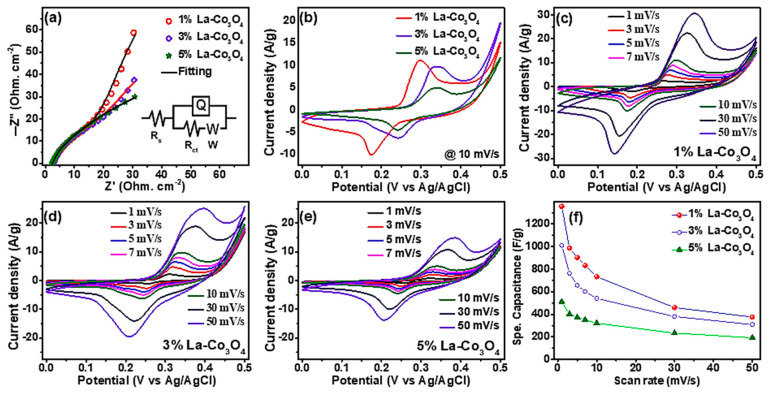
(**a**) EIS spectra of La-Co_3_O_4_ electrodes with 1–5% La content; (**b**) CV curves at a scan rate of 10 mVs^−1^ for 1 to 5% La-Co_3_O_4_ electrodes; (**c**–**e**) CV curves of (**c**) 1%, (**d**) 3%, and (**e**) 5% La-Co_3_O_4_ electrodes at scan rates of 1 to 50 mVs^−1^; and (**f**) variation in specific capacitance with scan rate for 1–5% La-Co_3_O_4_ electrodes.

Figure 7a shows the Nyquist plots of the La-doped Co_3_O_4_ electrodes. Each spectrum features a high-frequency intercept on the real axis, a depressed semicircle in the medium-frequency region, and an inclined line in the low-frequency region. The intercept corresponds to the solution resistance (*R*_s_ = 0.24 Ω), which reflects the combined effects of electrolyte, electrode, and contact resistances. The diameter of the semicircle represents the charge-transfer resistance (*R*_ct_) at the electrode/electrolyte interface. At the same time, the inclined line is associated with Warburg impedance, arising from ion diffusion processes within the porous electrode [55]. Among the samples, the 1% La-doped Co_3_O_4_ electrode shows the smallest semicircle diameter, indicating the lowest *R*_ct_ (2.2 Ω) and the most efficient charge-transfer kinetics. This improvement arises from enhanced conductivity and better electronic interactions at low La doping. However, higher doping levels (3% and 5%) increase *R*_ct_, likely due to structural distortions or blockage of electroactive sites caused by excess La incorporation. The inset equivalent circuit model (*R*_s_, *R*_ct_, a constant phase element *Q* for double-layer capacitance, and a Warburg element *W*) fits the experimental data well, supporting this interpretation. Overall, the EIS results confirm that 1% La doping achieves the best balance between conductivity and charge transfer, whereas higher doping concentrations degrade the electrochemical pathways. Figure 7b displays the CV profiles of the La-doped Co_3_O_4_ electrodes recorded at the scan rate of 10 mVs^−1^ within the potential window of 0 to 0.5 V (vs. Ag/AgCl). All electrodes show pronounced and nearly symmetric anodic and cathodic peaks, corresponding to the reversible Faradaic redox transitions of Co^2+^/Co^3+^ in an alkaline electrolyte according the following relationships [56,57]

(3) $${Co}_{3}O_{4}+{OH}^{-}+H_{2}O↔3CoOOH+e^{-},$$

(4) $$CoOOH+{OH}^{-}↔{CoO}_{2}+H_{2}O+e^{-}.$$

The 1% La-doped Co_3_O_4_ electrode delivers the highest peak currents and integrated CV area, indicating superior redox activity and faster kinetics compared with the 3% and 5% La-doped samples. At higher doping levels (3% La), partial blocking of electroactive sites and lattice distortions hinder the redox processes. Figure 7c–e presents the CV responses of the 1%, 3%, and 5% La-doped Co_3_O_4_ electrodes at scan rates in the range 1–50 mV s^−1^. As the scan rate increases, anodic peak potentials shift positively (from 0.26 to 0.35 V for 1% La; 0.30 to 0.39 V for 3% La; and 0.31 to 0.38 V for 5% La), while cathodic peaks shift negatively (from 0.19 to 0.13 V for 1% La, and 0.25 to 0.20 V for 3% and 5% La), consistent with polarization effects and internal resistance contributions [22,58]. Notably, the peak shapes remain well-preserved, indicating rapid electron/ion transport, as well as excellent reversibility. The specific capacitance (*C*_s_) was calculated from the CV curves using the relation [59,60]:

(5) $$C_{s}=\frac{\int_{V_{a}}^{V_{c}}I(V)dV}{mv\Delta V}$$

where $\int_{V_{a}}^{V_{c}}I(V)dV$ is the CV integral area, m is the active mass, *v* is the scan rate, and Δ*V* represents the potential window. Results are given in Table 2. As shown in Figure 7f and Table 2, the 1% La-doped Co_3_O_4_ electrode delivers the highest specific capacitance across all scan rates, followed by 3% and 5% La-doped Co_3_O_4_. A decrease in capacitance is observed with increasing scan rate for all electrodes, attributed to limited ion diffusion into the electrode’s deeper active sites at higher sweep rates [61]. These findings confirm that moderate La doping (approximately 1%) enhances electrochemical performance by promoting conductivity and redox kinetics, while excessive doping limits ion accessibility and compromises structural stability.

**Figure 8:**
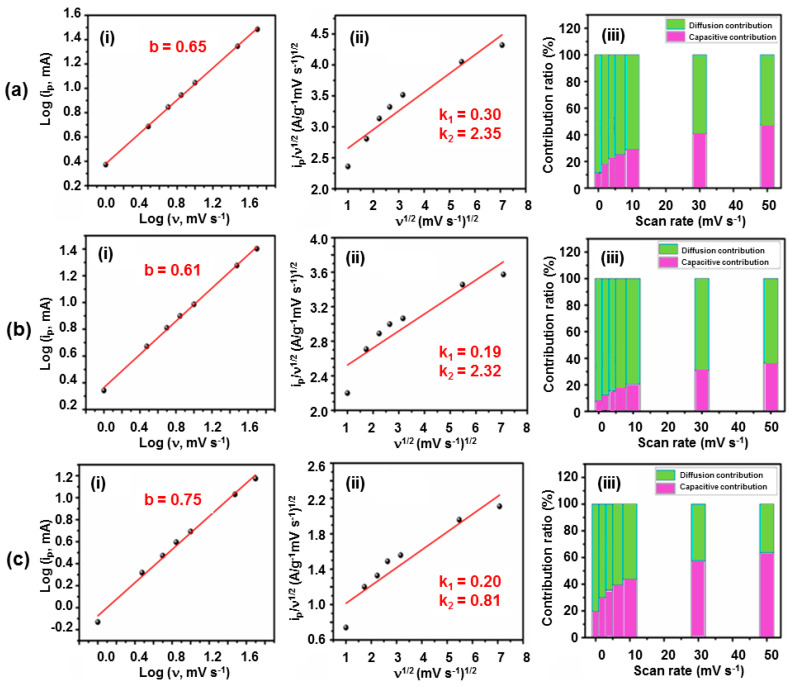
Determination of the diffusive and capacitive contributions for (**a**) 1%, (**b**) 3%, and (**c**) 5% La-doped Co_3_O_4_ electrodes with nanocubic morphology. (**i**) Plot of Log (*i*) vs. Log (*v*), (**ii**) plot of iv−12 vs.v12, and (**iii**) diffusion/capacitive contribution ratio vs. scan rate.

The charge–storage mechanism was further analyzed using the power–law relationship [55,62,63]:

(6) $$i_{p}=av^{b},$$

(7) $${\log i}_{p}=loga+b\log v,$$

where *i_p_* is the peak current, *v* is the scan rate, and *b* reflects the dominant mechanism (0.5—diffusion-controlled; 1.0—capacitive-controlled). The calculated *b*-values were 0.65 (1% La-Co_3_O_4_), 0.61 (3% La-Co_3_O_4_), and 0.75 (5% La-Co_3_O_4_) (Figure 8a–c), suggesting mixed capacitive- and diffusion-controlled processes in all samples. The contribution percentages are listed in Table S1 (Supplementary Materials file).

The capacitive and diffusion contributions were quantified using:

(8) iV= k1v+k2v12,

(9) i Vv−12=k1v12+k2.

where *k*_1_ν and *k*_2_ν^1/2^ represent the capacitive and diffusion-controlled contributions, respectively [59,63]. The capacitive and diffusion-controlled contributions in the electrodes at various scan rates were presented in Figure 8a(ii)–c(ii). This analysis reveals that the overall electrochemical performance of the La-doped Co_3_O_4_ electrodes is a result of the combined effects of both surface-controlled and diffusion-controlled processes. The relative dominance of these mechanisms varies with scan rate, as illustrated by the contribution plots in Figure 8a(iii)–c(iii). With increasing scan rate, a slight decrease in diffusion-controlled behavior is observed, indicating that surface-controlled redox reactions become more dominant under rapid charge–discharge conditions. The trend suggests that, at higher scan rates, ion intercalation into the bulk electrode is partially restricted due to the limited diffusion time, thereby favoring electrochemical reactions that occur at or near the electrode surface. At 50 mV s^−1^ scan rate, the capacitive contributions were calculated as 47.44% (1% La-Co_3_O_4_), 36.67% (3% La-Co_3_O_4_), and 63.58% (5% La-Co_3_O_4_). The 1% La-Co_3_O_4_ electrode demonstrates the most balanced contributions, enabling both rapid surface reflections and efficient ion intercalation.

**Figure 9:**
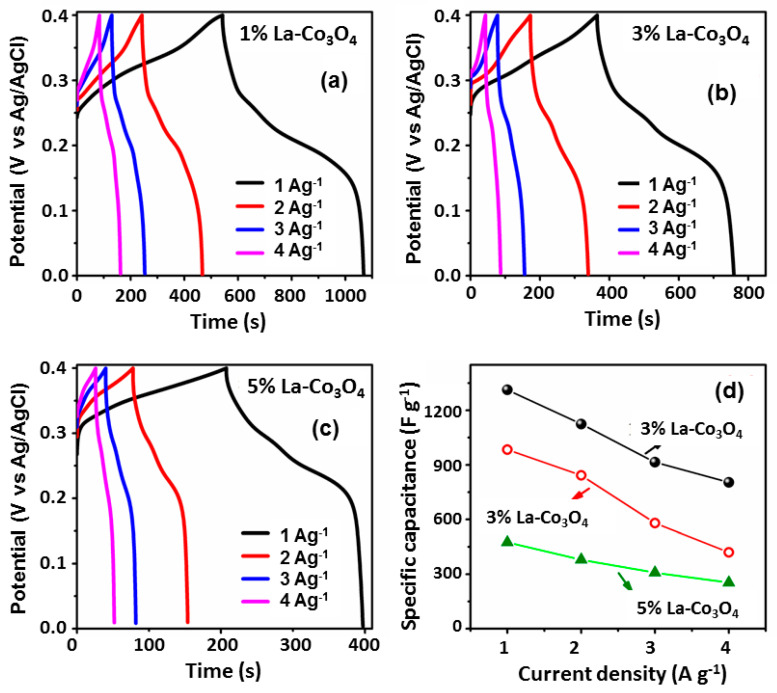
GCD curves of La-doped Co_3_O_4_ at different doping levels: (**a**) 1% La-, (**b**) 3% La-, and (**c**) 5% La-doped. (**d**) Variation in the specific capacitance with applied current densities for La-doped Co_3_O_4_ electrodes.

The potential versus time plots at constant applied currents (GCD curves) were utilized to measure the specific capacitance, rate capability, and cycling stability of the electrodes. Figure 9a–c shows the GCD plots of the 1% La-Co_3_O_4_, 3% La-Co_3_O_4_, and 5% La-Co_3_O_4_ electrodes at current densities ranging from 1 to 5 A g^−1^ in the potential window of 0 to 0.4 V. The shape of the charge and discharge profiles confirms the high reversible electrochemical behavior. Based on the discharge time (from 0.4 to 0 V), the specific capacitances of the electrodes were calculated by using the following equation [57,60]:

(10) $$C_{S}=\frac{I\;∆t}{m∆V},$$

where *I* represents the applied current, Δ*t* denotes the discharge time, *m* is the active mass of the electrode, and Δ*V* refers to the potential window. The calculated specific capacitances of the electrode at corresponding current densities are presented in Figure 9d. 

**Figure 10:**
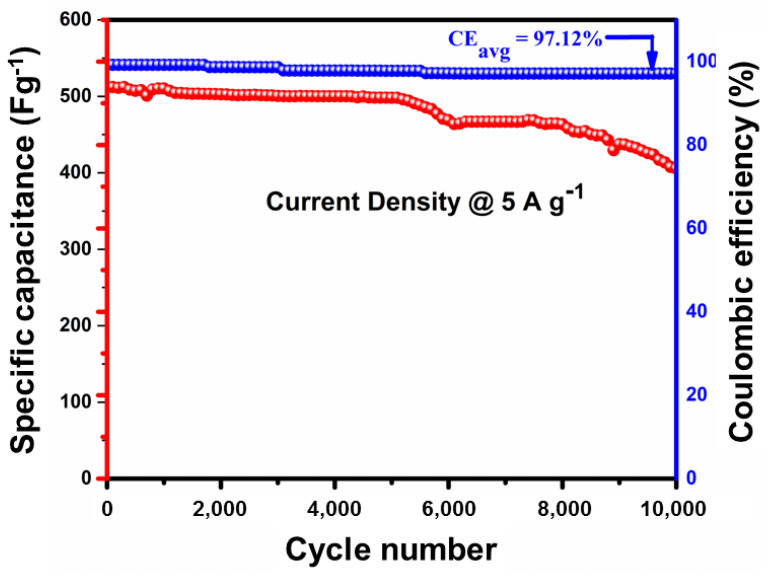
Cycle stability of the specific capacitance and Coulombic efficiency carried out at 5 A g^−1^ current density for the 1% La-doped Co_3_O_4_ nanocubes.

Among all electrodes, 1% La-Co_3_O_4_ exhibited the highest specific capacitances at all current densities, i.e., 1312, 1124, 915, and 804 F g^−1^ at current densities of 1, 2, 3, and 4 A g^−1^, respectively. Likewise, 3% La-Co_3_O_4_ delivered 984, 842, 579, and 420 F g^−1^, while 5% La-Co_3_O_4_ shows 474, 379, 308, and 253 F g^−1^ at the same current densities, respectively. The specific capacitance of all electrodes decreases with increasing applied current densities due to the limitation on the interaction between electrolyte ions and the electrode material at higher current densities. In addition, the cycling stability behavior of the high specific capacitance delivered sample (1%La-doped Co_3_O_4_) was recorded at 5 A g^−1^ for 10,000 cycles, as shown in Figure 10. It demonstrates a Coulombic efficiency of 97.12% along with 79.8% capacitance retention even after 10,000 cycles.

The electrochemical properties of the La-doped Co_3_O_4_ electrodes compare well with those of pristine Co_3_O_4_ (see Figures S2 and S3 showing CV, GCD, long-term cycling and EIS results).

The structural study shows considerable differences in crystallite size, dislocation density, and microstrain between pristine and La-Co_3_O_4_ composites. The 3% La-Co_3_O_4_ sample has the smallest crystallite size (17.5 nm), excellent dislocation density (3.27 × 10^15^ lines m^−2^), and maximum microstrain (0.0066 rd). Such structural distortions and lattice defects have been shown to produce extra active sites and increase electrolyte ion accessibility by forming high-energy grain boundaries and defect-mediated diffusion channels [64]. Electrochemical experiments reveal that the 1% La-Co_3_O_4_ electrode exhibits the maximum capacitance (1355 F g^−1^ at 1 mV s^−1^), outperforming both the 3% and 5% La-Co_3_O_4_ electrodes. This better performance is due to the optimal balance of crystallite size and lattice imperfections. Excessive defects, such as those found in the 3% La-Co_3_O_4_ sample, can hinder long-range crystallinity and reduce electrical conductivity, lowering overall capacitance [65]. However, the moderate microstrain and dislocation density increase the number of electroactive sites and promote the number of electroactive sites and fast Faradaic reactions. The 5% La-Co_3_O_4_ sample has the highest crystallite size (38.0 nm) and lowest dislocation density (0.692 × 10^15^ lines/m^2^), but has a lower capacitance (511 F g^−1^ at 1 mV s^−1^). This trend indicates that crystallite overgrowth and low defect density limit the number of accessible electroactive sites, thereby reducing ion/electron transport efficiency [66]. Furthermore, the capacitance of all electrodes decreases as the scan rate increases owing to diffusion restrictions and reduced ion penetration into the deeper porous networks. The tendency is particularly prominent in the 3% La-Co_3_O_4_ sample, corresponding with its significant microstrain, which may generate localized lattice distortions but inhibit effective electron transport under rapid charge–discharge circumstances. The 1% La-Co_3_O_4_ electrode preserves greater capacitance at higher scan rates, indicating that an intermediate defect density and moderate crystallite size offer a better balance of structural integrity, conductivity, and electrochemical activity. In conclusion, the correlation between microstructural characteristics and electrochemical performance highlights that controlled La incorporation into Co_3_O_4_ enables defect engineering, where moderate strain and dislocation density enhance electrochemical kinetics. In contrast, excessive or insufficient defect concentrations impact capacitive behavior.

Furthermore, the specific capacitance and cycling stability of the as-prepared La-doped electrode were compared with those of previously reported earth-rare-doped cobalt oxide electrodes, as presented in Table 3. The results demonstrate its superior electrochemical performance in a three-electrode system compared to the earlier cobalt oxide electrodes.

## 4. Conclusions

In summary, La-doped Co_3_O_4_ nanocubes were synthesized via a facile hydrothermal method, and the effects of La incorporation on their structural, morphological, and electrochemical properties were systematically studied. Structural analyses (XRD, Raman, and XPS) confirmed the successful substitution of La^3+^ into the Co_3_O_4_ lattice, introducing lattice strain, dislocations, and oxygen vacancies that modify the crystallinity and defect density of the spinel framework. Morphological observations revealed that low-level doping (1 mol%) preserved the nanocubic architecture, characterized by smooth surfaces and uniform particle distribution. In contrast, higher La concentrations (3–5 mol%) disrupted the growth process, leading to aggregation, surface roughening, and reduced structural order. Electrochemical investigations demonstrated that these structural differences strongly influence redox kinetics, conductivity, and charge storage. The 1% La-Co_3_O_4_ electrode achieved the highest specific capacitance (1312 Fg^−1^ at 1 Ag^−1^), fast ion/electron transport, as evidenced by a low charge transfer resistance, and remarkable durability, with 79.8% capacitance retention and 97.12% Coulombic efficiency over 10,000 cycles at 5 Ag^−1^. These results concluded that low La doping effectively balances crystallinity, defect engineering, and morphology control to maximize electrochemical activity, whereas excessive doping compromises structural integrity and hinders charge transport. Therefore, this study not only identifies 1% La-Co_3_O_4_ as an optimal electrode material for high-performance supercapacitors but also provides valuable insights into the role of controlled doping strategies in tailoring spinel-type oxides for next-generation energy storage systems.

## Figures and Tables

**Table 1:** Crystallite size, dislocation density, and microstrain of the pristine and La-doped (1–5%) Co_3_O_4_ samples.

Sample	Crystallite Size (nm)	Dislocation Density (Lines/m^2^)	Microstrain (rd)
pristine	30.6	1.060 × 10^15^	0.0033
1% La-Co_3_O_4_	32.4	0.954 × 10^15^	0.0036
3% La-Co_3_O_4_	17.5	3.273 × 10^15^	0.0066
5% La-Co_3_O_4_	38.0	0.692 × 10^15^	0.0030

**Table 2:** Specific capacitance of La doped Co_3_O_4_ samples at various scan rates.

Scan Rate (mV s^−1^)	Specific Capacitance (F g^−1^)		
	1% La-Co_3_O_4_	3% La-Co_3_O_4_	5% La-Co_3_O_4_
1	1355	1011	511
3	985	763	400
5	903	656	372
7	832	602	349
10	732	540	320
30	459	379	233
50	375	308	191

**Table 3:** Comparison of the electrochemical performance of the as-prepared La-doped Co_3_O_4_ electrode with other rare earth metal-doped (La, Nd, Gd, Sm, Ce, Pr, and Eu) cobalt oxides. Cycle numbers are marked in parenthesis.

Doping	Synthesis	Specific Capacitance	Stability	Ref.
Sm^3+^ (5.71 at%)	combustion	1016 F g^−1^ @ 5 mV s^−1^	93% (5000)	[34]
Gd^3+^ (5.46 at%)	combustion	764 F g^−1^ @ 5 mV s^−1^	-	[34]
La^3+^ (6.12 at%)	combustion	518 F g^−1^ @ 5 mV s^−1^	-	[34]
Nd^3+^ (5 mol%)	hydrothermal	1398 F g^−1^ @ 1 A g^−1^	95% (1000)	[67]
Pr^3+^ (2.79 at.%)	hydrothermal	99 F g^−1^@ 1 A g^−1^	88% (5000)	[68]
Ce^3+^	cation exchange	1288 F g^−1^ @ 2.5 A g^−1^	96.7% (6000)	[69]
La^3+^ (0.92 mol%)	precipitation	471 F g^−1^ @ 2 A g^−1^	93.3% (10,000)	[70]
Nd^3+^ (1.81 mol%)	precipitation	662 F g^−1^ @ 2 A g^−1^	93.5% (10,000	[70]
Eu^3+^ (3.71 mol%)	precipitation	1021 F g^−1^ @ 2 A g^−1^	91.8% (10,000)	[70]
Ce^3+^ (5 at%)	hydrothermal	1309 F g^−1^ @ 1 A g^−1^	90% (2000)	[31]
La^3+^ (1 mol%)	hydrothermal	1312 F g^−1^ @ 1 A g^−1^	79.8% @ 5 A g^−1^ (10,000)	this work

## Data Availability

All data are contained in this article.

## Supplementary Materials

The following supporting information can be downloaded at: https://www.mdpi.com/article/10.3390/nano15191515/s1, Figure S1: EDS spectrum for 1% La-doped Co_3_O_4_ powder; Figure S2: Electrochemical properties of the pristine Co_3_O_4_ electrode. (a) Cyclic voltammograms of recorded at various scan rates (5–100 mV s^−1^), (b) log (i_p_) vs. log (scan rate) graph, (c) GCD curves recorded at various current densities (1–4 A g^−1^), and (d) Cycling stability performance at a current density of 3 A g^−1^; Figure S3: Nyquist plot of the pristine Co_3_O_4_ electrode; Table S1: Contribution of capacitive- and diffusion-controlled processes in all La-doped Co_3_O_4_ samples.

## Abbreviations

The following abbreviations are used in this manuscript:

CV	cyclic voltammetry
DI	deionized
EDLCs	electric double-layer capacitors
EIS	electrochemical impedance spectroscopy
FESEM	field emission scanning electron microscopy
GCD	galvanostatic charge discharge
NMP	N-Methyl-2-pyrrolidone
PVDF	polyvinylidene fluoride
TMO	transition-metal oxide
XRD	X-ray diffraction
XPS	X-ray photoelectron spectroscopy
