# Supplementary material for: Lanthanum-Doped Co3O4 Nanocubes Synthesized via Hydrothermal Method for High-Performance Supercapacitors
Source: Nanomaterials (Basel). 2025 Oct 3;15(19):1515. doi: 10.3390/nano15191515 (PMC12526279; doi:10.3390/nano15191515)
Supplement: Supplementary file 1 [file nanomaterials-15-01515-s001.zip › nanomaterials-3895527-supplementary.pdf]

Supplementary Materials

Lanthanum-Doped Co_3O_4Nanocubes Synthesized via Hydrothermal Method for High-Performance Supercapacitors

Boddu Haritha[1], Mudda Deepak [1], Merum Dhananjaya [1], Obili M. Hussain[1,*] and Christian M. Julien[2,*]

[1] Thin Films Laboratory, Physics Department, Sri Venkateswara University, Tirupati 517502, India.
[2] Institut de Minéralogie, de Physique des Matériaux et Cosmologie (IMPMC), Sorbonne Université, UMR-CNRS 7590, 4 place Jussieu, 75252 Paris, France.
* Correspondence: hussainsvu@gmail.com(O.M.H.); christianjulien716@gmail.com (C.M.J.)

1. EDS Analysis of La-doped Co_3O_4

Energy dispersive X-ray spectroscopy (EDS) analysis confirms the presence of cobalt, oxygen, and lanthanum as constituent elements in the hydrothermally synthesized lanthanum doped Co_3O_4 nanocubes with cobalt (26.59 at.%), oxygen (71.45 at.%) and lanthanum (1.96 at%) present in a stoichiometric ratio consistent with the spinel cobalt oxide (Co_3O_4) phase. The elemental mapping also confirms the uniform distribution of cobalt and oxygen throughout the scanning area of the nanocubes.

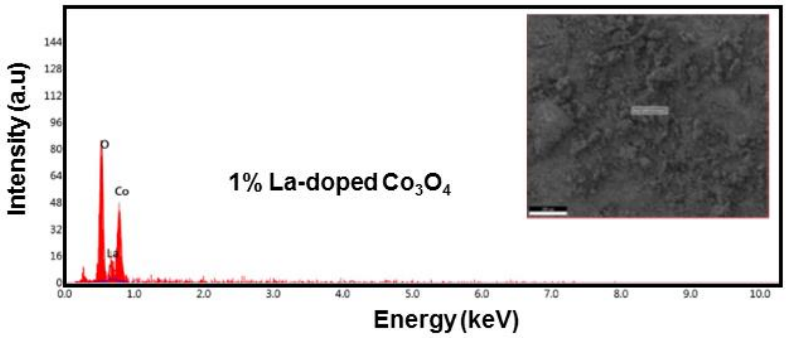

Figure S1. EDS spectrum for 1% La-doped Co_3O_4 powder.

2. Electrochemical properties of pristine Co_3O_4

The electrochemical performance of the synthesized pristine Co_3O_4 electrode was systematically evaluated using CV, GCD, and EIS analysis in a 3 mol L^{-1} aqueous potassium hydroxide (KOH) electrolyte at ambient temperature. Figure S2a illustrates the cyclic voltammetric response of the prepared electrode samples at different scan rate (5–100 mV s^{-1}) within a potential window of 0 to 0.5 V. The tested electrode exhibited a distinct pair of redox peaks, an oxidative peak around 0.4 V and reduction peak near 0.3 V with significantly larger integral area corresponding to the reversible redox transition between Co^{2+} and Co^{3+} oxidation states during the electrochemical reactions. Figure 2b presents the log (i_P) vs. log (scan rate) graph, from which the *b* parameter of the power-law relationship $i_P = av^b$ is determined to be 0.62 indicating a pseudocapacitive mechanism.

The calculated specific capacities of the pristine electrode were 440, 306, 210, 136, 104, 85, and 71.5 C/g at a scan rate of 5, 10, 20, 40, 60, and 80 mV s^{-1}. The GCD curves shown in Figure S2c exhibit voltage plateaus at ~0.4 and ~0.3 V versus Ag/AgCl, consistent with the redox peaks observed in the CV curves. The non-linear shape of the GCD profiles confirmed the battery-type electrochemical behavior of the electrodes. Figure S2d shows the cycling stability performance of the Co_3O_4 electrode at a current

density of 3 A g^{-1}. After 5000 cycles, the specific capacity retention is 93.67%, and after 10000 cycles, it retains 88% of its initial value, demonstrating exceptional long-term stability and durability.

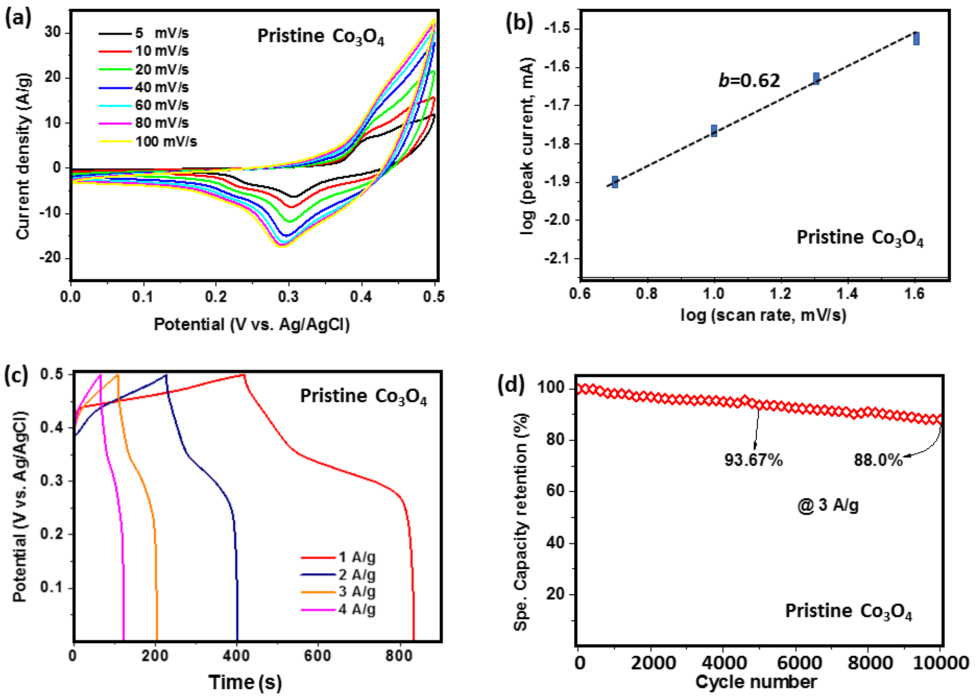

Figure S2. Electrochemical properties of the pristine Co_3O_4 electrode. (**a**) Cyclic voltammograms of recorded at various scan rates (5-100 mV s^{-1}), (**b**) log (i_p) vs. log (scan rate) graph, (**c**) GCD curves recorded at various current densities (1-4 A g^{-1}), and (**d**) Cycling stability performance at a current density of 3 A g^{-1}.

The Nyquist plots (Figure S3) revealed two distinct regions: a high-frequency semicircle and a low-frequency linear tail. The linear portion at low frequencies indicates the ion diffusion impedance (Warburg impedance, W). The Nyquist plots were well-fitted to an equivalent circuit model R(CR)(QR)(CR) (inset in Figure S3 with χ^2 values of 2.38×10^{-3}). The R_s and R_{ct} values for the Co_3O_4 electrode are 0.37 and 0.38 Ω, respectively, indicating the superior electrochemical conductivity. Furthermore, the steep linear segment in the Nyquist plot of Co_3O_4 confirmed its excellent ion-diffusion properties.

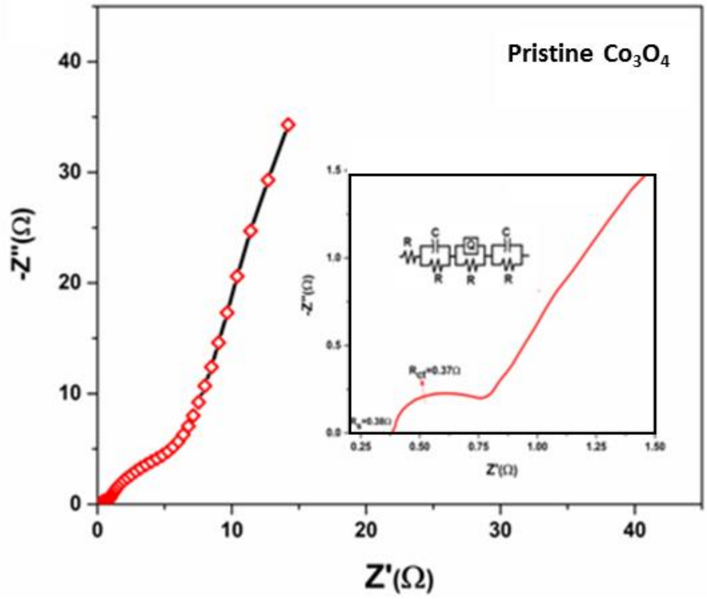

Figure S3. Nyquist plot of the pristine Co_3O_4 electrode.

Table S1. Contribution of capacitive- and diffusion-controlled processes in all La-doped Co_3O_4 samples.

Scan rate (mV s^{-1})	1%La-Co_3O_4		3%La-Co_3O_4		5%La-Co_3O_4	
	Capacitive	Diffusion	Capacitive	Diffusion	Capacitive	Diffusion
1	11.5	88.5	5.0	95.0	10.3	89.7
2	18.2	81.8	12.1	87.9	21.0	79.0
3	22.7	77.3	15.4	84.6	35.6	64.4
5	25.5	74.5	17.3	82.7	39.5	60.5
10	26.7	73.3	20.5	79.5	43.6	56.4
30	41.1	58.9	34.9	65.1	47.3	52.7
50	47.4	52.6	36.7	63.3	63.6	36.4
